# Localized Autoimmune Pancreatitis: Report of a Case Clinically Mimicking Pancreatic Cancer and a Literature Review

**DOI:** 10.1097/MD.0000000000001656

**Published:** 2015-10-23

**Authors:** Zhe Cao, Rui Tian, Taiping Zhang, Yupei Zhao

**Affiliations:** From the Department of General Surgery, Peking Union Medical College Hospital, Chinese Academy of Medical Sciences, Beijing, China; and Peking Union Medical College, Beijing, China.

## Abstract

Autoimmune pancreatitis (AIP) is a rare disease with clinical presentations that greatly mimic pancreatic cancer (PC). It is critical for clinicians to distinguish AIP from PC because their treatments and prognoses are entirely different. Typical images show characteristic features such as diffuse pancreatic swelling and strictures of the main pancreatic duct (MPD). However, AIP may present as a localized pancreatic mass, in which case it is very difficult to differentiate from PC. Here, we report a case of a 40-year-old man with computed tomography (CT) imaging studies confirming an area of low-density neoplasm in the uncinate process of the pancreas with dilation in the common biliary duct (CBD) and MPD. Increased uptake in the uncinate mass was observed by positron emission tomography (PET)/CT scan, which strongly suggested PC. Further laboratory analyses showed a marked elevation of serum IgG4. Because there was not enough evidence to rule out a diagnosis of malignancy, a histopathological biopsy became the criterion standard. An endoscopic ultrasound (EUS)-guided needle biopsy failed. As an alternative, a pancreaticoduodenectomy was conducted for the biopsy, and pathological analysis confirmed IgG4-related sclerotic chronic pancreatitis with moderate lymphoplasmacellular infiltration.

We suggest that an accurate preoperative diagnosis for localized AIP with MPD and CBD obstructions mimicking PC is of great importance. Radiological imaging findings, particularly observations of diffused enlargement of the pancreas and delayed enhancement during the venous and portal phases, are essential for diagnosing AIP. Careful consideration should be given if serum IgG4 was taken as a special indicator for a differential diagnosis between AIP and PC. A history of IgG4-related diseases involving the biliary, lacrimal, salivary, retroperitoneal, renal, or pulmonary systems should also be highlighted. Thus, the pathology of extrapancreatic organs can be utilized as diagnostic evidence when the pathology for a pancreatic mass is not available, as in the case presented here. Furthermore, cautious use of hormone therapy is indicated for patients who cannot be ruled out as having PC. The results of future studies on localized AIP are eagerly awaited.

## INTRODUCTION

Autoimmune pancreatitis (AIP), regarded as a pancreatic manifestation of IgG4-related diseases, is a rare disease that clinically mimics pancreatic cancer (PC). It is very important for clinicians to distinguish between AIP and PC because the treatments and prognoses are significantly different. Although the diagnosis of AIP has been improved through a growing awareness and proposed diagnostic criteria,^1,2^ there remains no practical strategy to differentiate between AIP and PC, particularly localized AIP.

Here, we report a rare case of a patient with localized mass-forming AIP strongly suspected of PC because of MPD and CBD obstructions shown on a computed tomography (CT) scan.

## CASE REPORT

**FIGURE 1:**
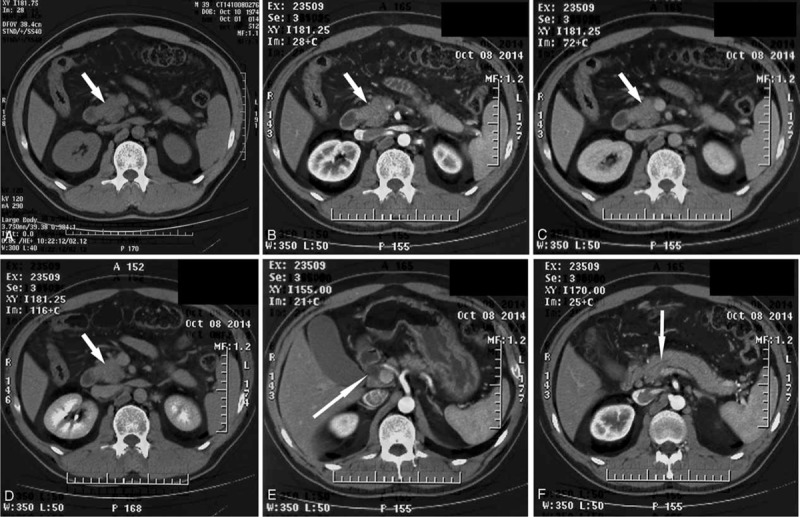
Abdominal CT demonstrates a low-density mass measuring 3.5 × 3.2 cm in the uncinate process of the pancreas, with dilated common bile and pancreatic ducts. (A) to (D) represent the plain phase, arterial phase, portal phase, and venous phase, respectively. (E) shows the dilated bile duct, and (F) reveals the dilated pancreatic duct.

**FIGURE 2:**
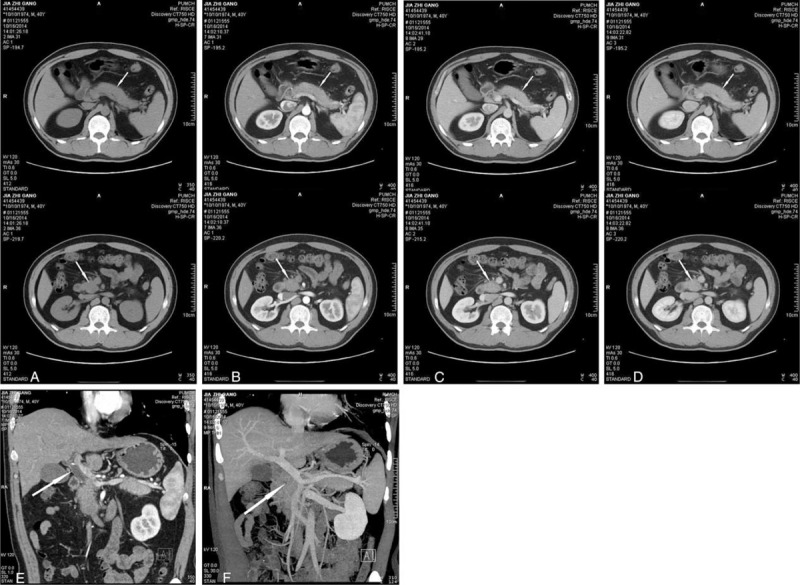
Pancreatic protocol with CT + 3D reconstruction demonstrates an uncinate mass of the pancreas and the swollen in the body and tail of the pancreas. (A) to (D) represent the plain phase, arterial phase, portal phase, and venous phase, respectively. (E) shows the dilated common bile duct, and (F) reveals the anatomical relationship of the uncinate process and the superior mesenteric vein.

**FIGURE 3:**
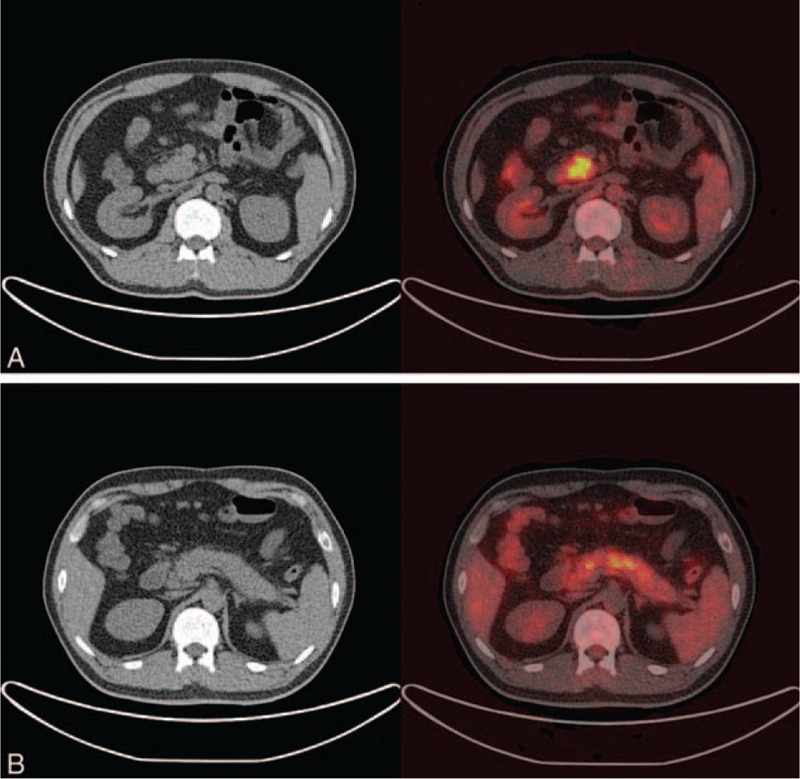
Positron emission tomography/computed tomography body imaging demonstrates (A) a swollen uncinate process of the pancreas with increased uptake, which is suspicious for malignancy, and (B) diffusely increased uptake in the body of pancreas, which is suspicious for secondary inflammation.

A 40-year-old man presented at a local hospital with upper abdominal pain for 20 days, as well as jaundice and kaolin stools for 10 days, before his clinic visit on September 29, 2014. At that time, liver enzymes were checked and found to be elevated, with total bilirubin (TBIL) 62.8 μmol/L (normal range 5.1–22.2 μmol/L), direct bilirubin (DBIL) 49.7 μmol/L (normal range 0.0–6.8 μmol/L), glutamyl transpeptidase (GGT) 999.9 U/L (normal range 10–60 U/L), alkaline phosphatase (ALP) 207 U/L (normal range 15–40 U/L), aspartate aminotransferase (AST) 335.1 U/L (normal range 15–40 U/L), and alanine aminotransferase (ALT) 335.1 U/L (normal range 9–50 IU/L). Serum tumor markers fluctuated within normal ranges at CA19–9 17 U/mL and CEA 1.2 ng/mL. Both an abdominal ultrasound and a CT scan indicated a localized space-occupying lesion in the pancreas, with dilated biliary and pancreatic ducts, suspicious for malignancy (see Fig. 1). He suffered a 4-kg weight loss during the course of the disease. The diagnosis was “suspected malignant neoplasm in the head of the pancreas.” He denied a history of diabetes mellitus or biliary diseases. Three years prior, he underwent surgical treatment for “an occupying lesion in the left lacrimal gland” and stated that the postoperative pathology confirmed the lesion to be benign. He was referred to our outpatient clinic on October 15, 2014 and was admitted for obstructive jaundice and an occupying lesion in the pancreas head. Liver enzymes were rechecked, with TBIL 109 μmol/L (normal range 5.1–22.2 μmol/L), DBIL 62.5 μmol/L (normal range 0.0–6.8 μmol/L), GGT 989 U/L (normal range 10–60 U/L), ALP 318 U/L (normal range 15–40 U/L), AST 123 U/L (normal range 15–40 U/L), and ALT 288 U/L (normal range 9–50 IU/L). Serum tumor markers fluctuated within normal ranges at CA19–9 19.1 U/mL and CEA 1.93 ng/ml. A contrast-enhanced CT with combined TLC scanning and 3-dimensional reconstruction was applied, and the image was characterized by a low-density mass anatomically close to the superior mesenteric vein in the uncinate process of the pancreas with a dilated common biliary duct. The body and tail of the pancreas were observed to be slightly swollen with diminished lobules (see Fig. 2). A positron emission tomography (PET)/CT scan indicated swelling in the uncinate process of the pancreas, and increased glucose metabolism was observed with a maximum standardized uptake value (SUV) of 3.8. Lymphatic metastasis was also noticed, indicating a higher risk of malignancy, and a diffused elevation of metabolic levels in the pancreas body was observed as a secondary infection (Fig. 3).

The patient received liver-protecting therapy (Essentale, Aventis Pharma, Waltloo, Pretoria, South Africa), and his jaundice was significantly relieved. Serum IgG4 was then examined and found to be 10,500 mg/L. To define the nature of the lesion in the uncinate process of the pancreas, an endoscopic ultrasound (EUS)-guided needle biopsy was attempted. However, the patient could not tolerate EUS, and, thus, the EUS-guided needle biopsy harvested no tissue from the lesion. These findings were explained to the patient, informing him that a mass in the uncinate process might be AIP but that malignancy could not be ruled out. A plan for surgical exploration was proposed, with the possibility of a pancreaticoduodenectomy for benign lesions, and repeating the needle biopsy was recommended for greater clinical benefit, but the patient rejected both options. He was discharged with a temporary diagnosis of “pancreatic uncinate mass with unclear pathology,” and further visits to the Gastroenterology Outpatient Clinic were required.

**FIGURE 4:**
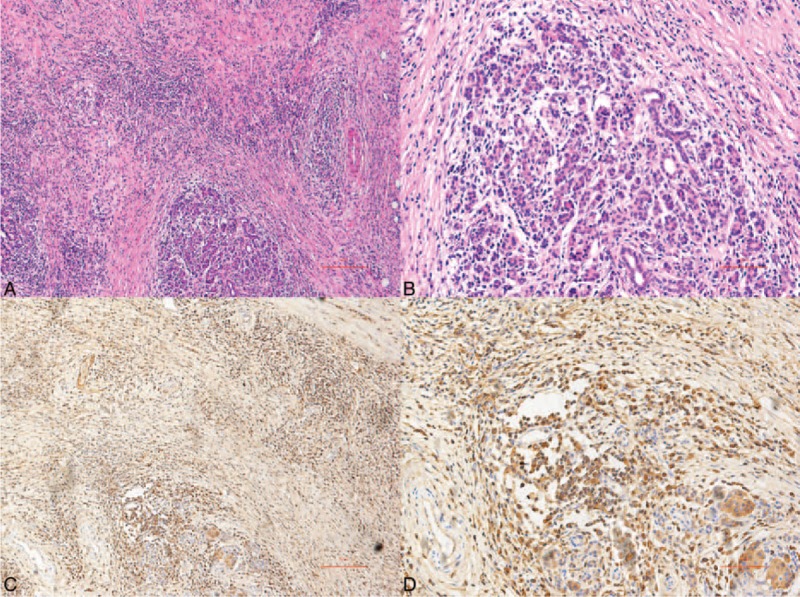
Histopathologically, this case was characterized as IgG4-related sclerosing chronic pancreatitis. (A) Hematoxylin and eosin staining (40×), (B) hematoxylin and eosin staining (100×), (C) significant infiltration of IgG4-positive cells (40×), (D) significant infiltration of IgG4-positive cells (100×).

Six days after leaving the hospital, the patient returned with recurrence of abdominal pain, jaundice and kaolin stools, as well as a weight loss of 11 kg. He then agreed to accept surgical treatment, without reference to the uncinate mass being benign or malignant. In this case, PD was performed under general anesthesia. Intraoperative findings showed a hard mass measuring approximately 3.0 cm in the uncinate process and a hard distal pancreas, indicating obstructive chronic pancreatitis. A postoperative pathologic examination suggested IgG4-related sclerotic chronic pancreatitis with moderate lymphoplasmacellular infiltration (Fig. 4), and a diagnosis of AIP was confirmed. There was no severe infection, fistula, or other surgical complications, and the patient was discharged 14 days after surgery. After 7 months of follow-up, the patient reported no discomfort, such as fever or abdominal pain.

## DISCUSSION

AIP is a type of rare chronic pancreatitis characterized by an autoimmune inflammatory process. The overall prevalence of this disease is 0.9 in every 100,000 individuals, which accounts for 2% to 10% of all patients with chronic pancreatitis. AIP occurs in both sexes, but it presents twice as often in men than in women.^3^ Patients with AIP mainly present with abdominal pain and obstructive jaundice, similar to PC, and localized AIP is the most common benign suspicion of PC and usually leads to Whipple's disease. Nevertheless, AIP lesions respond rather readily to corticosteroids, leading to rapid and sustained relief of the pancreatic mass, obstructions in the biliary duct, and strictures in the pancreatic duct. Therefore, a differential diagnosis between AIP and PC is of great importance.

Since the term “autoimmune pancreatitis” was introduced by Yoshida et al in 1995, great interest has been focused on this disease. In 2002, the Japan Pancreas Society (JPS) for the first time formulated the diagnostic criteria for AIP. Subsequently, Korea and the United States also put forward their criteria.^4,5^ Integrated diagnostic criteria were fostered by the International Association of Pancreatology (IAP) through an international consensus in 2010, featuring criteria for imaging, laboratory results, extrapancreatic organ involvement, histopathology, and treatment.^6^ According to these criteria, typical radiological imaging findings, in particular the observation of a diffused and localized enlargement of the pancreas and strictures in MPD, are essential for diagnosing AIP. However, localized AIP, which can mimic pancreatic adenocarcinoma, remains difficult to differentiate from PC.

In our case, the patient presented with abdominal pain and jaundice, and images revealed a mass in the uncinate process of the pancreas, with dilated bile and pancreatic ducts. Increased uptake in the uncinate mass was observed with a PET/CT scan, pointing to a diagnosis of PC. At this point, a surgical attempt to obtain pathology would be reasonable. However, when we review this case, the following questions deserve special attention:

CT is regarded as an important imaging approach in a differential diagnosis between AIP and PC. Typical CT features of AIP include diffused allantoid enlargement of the pancreas and a radiolucent ring around the pancreas. In this case, localized AIP presented as a low-density mass, making it difficult to distinguish it from PC, with the exception that AIP shows delayed homogenous enhancement during the portal and venous phases. Other studies have been performed on this topic.^7^ One retrospective study reported that localized AIP with a CT value ≥28Hu during the delayed phase showed better sensitivity at 87.5% and specificity at 100%. This conclusion remains to be clarified because it was a retrospective study using a small sample; however, the above study indicates that delayed homogenous enhancement can be a differential point between localized AIP and PC.

Careful consideration must be given if serum IgG4 was taken as a special indicator for a differential diagnosis between AIP and PC. This is because >20% of all AIP patients have normal IgG4 levels, whereas 7% to 10% of PC patients have elevated IgG4 levels. Moreover, the prevalence of AIP is much lower than PC, and serum IgG4 used to have a poor positive predictive value for AIP. In all, serologic elevation of IgG4 alone is not sufficient for an AIP diagnosis.

The significance of PET/CT scans in a differential diagnosis between AIP and PC remains to be examined. Nanni et al^8^ thought poorly of the role that PET/CT plays in differentiating between AIP and malignancy, for active AIP always shows increased uptake, similar to malignancy. However, increased uptakes are observed in salivary glands, submandibular glands, kidneys, and other extrapancreatic organs in some patients, which help differentiate between AIP and malignancy. However, a PET/CT scan can act as an assessment for corticosteroid therapy on AIP.

Cautious application should be used for diagnostic corticosteroid treatments. Some physicians tend to use diagnostic corticosteroid treatments when AIP is suspected. However, pancreatic lymphoma and even some subtypes of PC also respond to corticosteroid therapy.^9^ According to the experience at our hospital, this decision should not be made until the possibility of PC is excluded. The effectiveness of corticosteroid treatment alone cannot serve as a diagnostic basis. For this patient, a low-density mass in the uncinate process could not be exempted from malignancy before surgery, and swelling in the body and tail of the pancreas could be interpreted as pancreatitis that resulted from an obstruction in the pancreatic duct. Hence, early corticosteroid therapy without a clear pathology is not recommended.

AIP is the pancreatic manifestation of IgG4-related diseases, and the potential target organs involved include the biliary, lacrimal, salivary, retroperitoneal, renal, and pulmonary systems. These tissues share the general trait of specific infiltration of IgG4-positive cells, which could serve as a diagnostic basis in biopsy pathology and increase diagnostic accuracy. In this case, although a pancreatic biopsy could not be obtained due to the patient's intolerance to the EUS-guided needle biopsy, the patient had undergone surgical treatment for “an occupying disease in the left lacrimal fossa” 3 years prior. After the surgery, we made a detailed pathological inquiry to a local hospital, and the detailed description we received was “lymphadenosis, with significant infiltration of IgG4-positive cells.” This taught us a lesson that for patients for whom AIP is suspected, the involvement of those extrapancreatic organs should be addressed. Moreover, the pathology of extrapancreatic organs can be utilized as diagnostic evidence when the pathology for the pancreatic mass is not available. Once a correct diagnosis is established, appropriate treatment can be applied, and rapid and sustained resolution of extrapancreatic symptoms and improvement in pancreatic images are indicators of the therapeutic effect. However, it must be noted that extrapancreatic symptoms may not always parallel the severity of the pancreatic lesion.

When the pancreatic neoplasm is located in the uncinate process, it might be difficult to acquire a pathological diagnosis with EUS-guided needle biopsy. Therefore, an intraoperative biopsy can be an option. Once malignancy has been pathologically confirmed, there are indications for surgical removal of the lesion. However, for cases that are highly suspicious for malignancy before surgery, such as this case, whereas none of intraoperative findings supported malignancy, the necessity of surgery on such cases and the best choice of surgical method remain controversial.

In conclusion, AIP is a pancreatic disease with a relatively low prevalence compared with PC. Although AIP is a benign disease, it is commonly misdiagnosed as a malignancy. Clinicians should increase their awareness of AIP to avoid further misdiagnoses of cancer and unnecessary surgery. The results of future studies in this area are eagerly awaited.
